# The dog as a naturally-occurring model for insulin-like growth factor type 1 receptor-overexpressing breast cancer: an observational cohort study

**DOI:** 10.1186/s12885-015-1670-6

**Published:** 2015-10-08

**Authors:** Laetitia Jaillardon, Jérome Abadie, Tiffanie Godard, Mario Campone, Delphine Loussouarn, Brigitte Siliart, Frédérique Nguyen

**Affiliations:** 1 Oniris, Université Nantes-Angers-Le Mans, Department of Human Health, Biomedical Research and Animal Models, AMaROC Unit and LDHvet laboratory, Nantes Atlantic College of Veterinary Medicine, Food Science and Engineering, Site de la Chantrerie, Route de Gachet, Nantes, F-44307 France; 2 Institut de Cancérologie de l’Ouest, Boulevard Jacques Monod Saint Herblain-Nantes cedex, Centre de Recherche du Cancer Nantes-Angers, UMR-INSERM U892/CNRS 6299, Nantes, F-44805 France; 3 Hopital G&R Laënnec, Boulevard Jacques Monod, Saint Herblain-Nantes cedex, Nantes, F-44093 France

**Keywords:** Spontaneous animal model, Canine mammary carcinoma, IGF1R, Triple-negative, Comparative oncology

## Abstract

**Background:**

Dogs spontaneously develop invasive mammary carcinoma with a high prevalence of the triple-negative (TN) subtype (lack of ER-Estrogen Receptor and PR-Progesterone Receptor expression, lack of HER2-Human Epidermal Growth Factor Receptor 2 overexpression), making this animal model relevant for investigating new therapeutic pathways. Insulin-like growth factor Type-1 receptor (IGF1R) is frequently overexpressed in primary human breast cancers, with a growing role in the TN phenotype. The purpose of this study was to investigate the Dog as a candidate model for IGF1R-overexpressing mammary carcinoma.

**Methods:**

150 bitches with canine mammary carcinoma (CMC) and a known 2-year follow-up were retrospectively included. IGF1R expression was assessed by immunohistochemistry (IHC) using a similar scoring system as for HER2 in breast cancer. The prognostic value of the IGF1R expression was assessed in terms of overall and specific survival as well as disease-free interval (DFI).

**Results:**

47 CMC (31 %) were classified as luminal and 103 (69 %) as triple-negative (TN-CMC). 41 % of CMC overexpressed IGF1R (IHC score 3+) of which 76 % were TN-CMC and 62 % grade III. IGF1R overexpression was associated with aggressive features including lymphovascular invasion, histological grade III, low ER expression and the TN phenotype. Univariate and multivariate analyses revealed that IGF1R overexpression was associated with shorter overall and specific survivals and shorter DFI in TN-CMC.

**Conclusions:**

IGF1R overexpression is common and related to a poor outcome in canine invasive mammary carcinoma, particularly in the triple negative subtype, as in human breast cancer. Preclinical studies using the Dog as a spontaneous animal model could be considered to investigate new therapies targeting IGF1R in triple-negative breast cancer.

**Electronic supplementary material:**

The online version of this article (doi:10.1186/s12885-015-1670-6) contains supplementary material, which is available to authorized users.

## Background

The identification of relevant naturally-occurring animal models is of particular interest in oncology in order to accelerate the development of effective diagnostic and therapeutic innovations for human patients. The Dog is a really good candidate as its physiology [1] and genome [2] are very similar to that of humans. Dogs share the same environment as humans with highly comparable nutritional needs, and naturally develop various cancers with a shorter natural history [3]. This spontaneous animal model could be highly beneficial to translational breast cancer research as the human classification of breast cancers is relevant to canine mammary carcinomas [4, 5], even if some histological entities (particularly complex mammary carcinoma) are quite different between human and dog [6]. Interestingly, the triple negative (TN) immunophenotype, one of the most aggressive breast cancer subtypes defined by the lack of ER (Estrogen Receptor), PR (Progesterone Receptor) and HER2 (Epidermal Growth factor Receptor type 2) overexpression, is well recognized in dogs [7, 8].

In various human cancers including breast cancer, the Insulin-like Growth Factor (IGF) family is closely related to oncogenesis [9, 10], *in situ* tumor growth [11], invasion and metastasis [11], with IGF1R (Insulin-like Growth Factor Type 1-Receptor) acting as a real oncogene and being overexpressed in more than 50 % of primary breast cancers [12]. This is particularly true for the TN breast cancer cells (estrogen-unresponsive), in which IGF1R is largely expressed and IGF-1 stimulates proliferation and survival, making them responsive *in vitro* to anti-IGF1R therapies [13, 14]. An ongoing phase I clinical trial of the IGF1R inhibitor OSI-906 in humans affected by advanced solid tumors showed few adverse effects and no unexpected toxicities [15]. Even if a phase II clinical trial using ganitumab (an anti-IGF1R antibody) did not show any improvement for women with hormone-receptor positive and advanced breast cancer [16], a phase I trial using another anti-IGF1R antibody (cixutumumab) showed promising results by prolonging stable diseases [17]. IGF1R expression is highly related to prognosis in breast cancer, with a prognostic value dependent on the ER status of the tumors: in ER-positive breast cancer, IGF1R overexpression is related to favorable outcome [18] as opposed to ER-negative carcinomas, in which IGF1R overexression is associated with a poor outcome [19].

In canine mammary carcinoma, tissue GH (Growth Hormone) and IGF-1 have been positively correlated with tumor malignancy, as well as with tissue levels of progesterone and 17β-estradiol [20]. IGF1R expression has also been reported to be higher in histologic types of worse prognosis [21] although some studies did not show any significant association between IGF1R expression in mammary carcinomas and the clinical outcome in canine patients [22]. In addition, IGF-1 and IGF1R have been implicated in other canine cancers including osteosarcoma [23, 24], malignant melanoma [25] and testis tumors [26], suggesting a major role of the IGF system in canine oncology.

In this study, IGF1R expression was retrospectively investigated by immunohistochemistry (IHC) in a large cohort of canine invasive mammary carcinomas in order to determine the extent of similarities between canine and human mammary carcinomas, with respect to the role of IGF1R in tumor biology and natural history.

## Methods

### Patients and samples

Invasive mammary carcinomas surgically removed from 150 bitches, formalin-fixed and sent to two laboratories of veterinary histopathology (Laboratoire d’Histopathologie Animale, Oniris, Nantes, France and Laboratoire d’Anatomie Pathologique Vétérinaire d’Amboise, Amboise, France) between 2007 and 2010 were retrospectively selected. The owners’ written consent and approval from the Oniris College of Veterinary Medicine local Animal Welfare Committee were obtained prior to inclusion.

Dogs were eligible for inclusion when a diagnosis of invasive mammary ductal carcinoma was established by histological analysis and confirmed by an absent layer of p63-positive myoepithelial cells (anti-p63 antibody, clone ab111449, abcam plc) by immunohistochemistry (IHC) that differentiates invasive from *in situ* breast ductal carcinoma [27, 28]. All female dogs that had received any adjuvant chemotherapy and/or for which follow-up was not available for at least 2 years after mastectomy, were excluded from the study.

Breed, age and reproductive status (including age of neutering) at time of mastectomy, as well as the number and location of mammary carcinoma(s), were recorded for each bitch. Two-year follow-up was obtained through telephone interviews with referral veterinarians with particular emphasis on the occurrence of recurrence (i.e. the occurrence of an another mammary tumor on the same mammary gland) and/or of a new primary mammary tumor, and the animal’s outcome (alive or dead and cause of death, i.e., unrelated or related to the mammary carcinoma whether the animals died naturally or were euthanatized because of metastases). Overall Survival (OS) was defined as the time between surgery (mastectomy) and death from any cause; uncensored cases corresponded to dead animals; censored cases were still alive at least two years post-diagnosis. Specific Survival (SS) was defined as the time between surgery and death attributable to the mammary carcinoma; censored cases corresponded to dogs still alive, dogs that died from unknown cause, and dogs that died from another cause than the mammary carcinoma. The interval from surgery to the first local recurrence, new primary tumor, lymph node metastasis and/or distant metastasis was also assessed, and defined the disease-free interval (DFI).

### Histopathology and immunohistochemistry (IHC)

All tumors were paraffin-embedded immediately after reception. 4 μm-thick serial sections were performed onto positively charged slides (Superfrost plus, Menzel-Glaser, Germany). After Hematoxylin and Eosin (HE) staining, mammary carcinomas were classified by five independent pathologists (one human breast pathologist and four veterinary pathologists) according to the WHO’s classification system of canine mammary tumors [28, 29, 30], and graded according to the criteria of Elston and Ellis [31] as well-differentiated (grade I), moderately differentiated (grade II) or poorly differentiated (grade III) carcinomas. The histologically assessed size of mammary carcinoma(s) with 2 cm chosen as a threshold according to the American Joint Committee on Cancer (AJCC), lymphovascular invasion, completeness of surgical excision, dermal infiltration, cutaneous ulceration, muscle invasion, squamous differentiation, inflammation and central necrosis were recorded for each case. In case of multifocal or multicentric carcinomas, the tumor with the highest pathologic size and/or highest histological grade was included in the study.

Automated IHC (Benchmark XT Ventana, Roche Diagnostics) was performed using antibodies against ERα (Estrogen Receptor alpha, clone C311, Santa Cruz), PR (Progesterone Receptor, clone 1E2, Ventana), HER2 (Human Epidermal Growth Factor Receptor 2 clone 4B5, Ventana), Ki-67 (clone MIB1, Dako), CK5/6 (Cytokeratin 5/6, clone D5/16B4, Dako), EGFR (Epidermal Growth Factor Receptor Type 1 clone 31G7, Invitrogen) and IGF1R (Insulin-like Growth Factor type 1-Receptor clone G11, Ventana). IHC protocols are detailed in Additional file 1: Table S1.

Scoring of the immunohistochemical staining was performed by the five independent pathologists. ER, PR and Ki-67 were assessed based on the number of positive nuclei among 500 counted cells (manual image analysis involving the use of the image J software, Research Service Branch, National Institute of Health, Bethesda, Maryland, USA). ER and PR were considered positive if nuclear staining was observed in more than 10 % of the cells [32] and Ki-67 in more than 20 % of the cells [33]. HER2 [32, 34] was scored as follow: 0 for no staining at all or incomplete, faint/barely perceptible membrane staining in less than 10 % of the cells; score 1+ for incomplete and faint/barely perceptible membrane staining in more than 10 % of the cells; 2+ for circumferential and incomplete and/or weak/moderate membrane staining in more than 10 % of the cells; and 3+ for circumferential and complete and intense membrane staining in more than 10 % of the cells. Carcinomas were considered positive for HER2 only for a 3+ IHC score [32]. IGF1R was scored in accordance with the HER2 expression scoring system [19, 35]: a negative result was defined as the complete absence of membrane staining (score 0) or the presence of weak membrane staining in less than 10 % of the cells or incomplete membrane staining in more than 10 % of the cells (score 1+) in any portion of the tumor; a score 2+ was applied for complete and weak to moderate membrane staining in more than 10 % of the cells; and a score 3+ for complete and intense membrane staining in more than 10 % of the tumor cells [34]. EGFR [36] was considered positive if membrane staining was observed in more than 10 % of the cells. Positivity to cytokeratins 5/6 (CK5/6) was defined with a threshold of 10 % [37].

Negative controls for IHC were included in each run, and consisted in replacing the primary antibody with normal mouse or rabbit serum (prediluted reagents, Roche Diagnostics). The positive controls were internal controls in most cases (i.e., skin epidermis and hair follicles for Ki-67, CK 5/6, EGFR and IGF1R; mammary gland surrounding the carcinoma for ER and PR), as stated in Table 1. For HER2 IHC, the pathway HER2 4-in-1 control slides (Roche Diagnostics) were chosen because they allow the quality of staining for each HER2 score (0, 1+, 2+, 3+) to be assessed.

**Table 1:** Characteristics of the dogs and their invasive mammary carcinomas

Parameters	Data
	n (%)
Total	150 (100)
Age in yrs	Median 11 yrs, Range [5.1–16.3 yrs]
5.1–10.9 yrs	73 (48.7)
≥11 yrs	77 (51.3)
Tumor size	
< 2 cm	53 (36.5)
≥ 2 cm	92 (63.5)
Histological type	
Squamous cell carcinoma	6 (4)
Simple carcinoma: Anaplastic	6 (4)
Complex carcinoma	11 (7.3)
Simple carcinoma: Solid	40 (26.7)
Simple carcinoma: Tubulopapillary	87 (58)
Histological grade (Elston & Ellis)	
Grade I	19 (12.6)
Grade II	58 (38.7)
Grade III	73 (48.7)
Lymph node status	
Positive (N1)	32 (21.3)
Negative (N0)	19 (12.7)
Unknown (NX)	99 (66)
ER expression	
Positive (≥ 10 %)	35 (23.3)
Negative (< 10 %)	115 (76.7)
PR expression	
Positive (≥ 10 %)	20 (13.3)
Negative (< 10 %)	130 (86.7)
HER2	
Score 0	85 (56.7)
Score 1+	50 (33.3)
Score 2+	15 (10)
Score 3+	0
CK5/6	
Positive (≥ 10 %)	89 (59.3)
Negative (< 10 %)	61 (40.7)
EGFR	
Positive (≥ 10 %)	72 (48)
Negative (< 10 %)	78 (52)
Immunophenotype	
Luminal-A	17 (11.3)
Luminal-B	30 (20)
Triple-negative basal like	70 (46.7)
Triple-negative non basal like	33 (22)
IGF1R expression	
Score 0–1+	34 (22.7)
Score 2+	54 (36)
Score 3+	62 (41.3)
Survival Time in days	Median 331 days, Range [2–2608]

*yrs* years, *ER* Estrogen Receptor, *PR* Progesterone Receptor, *HER2* Epidermal Growth Factor Receptor 2, *CK5/6* Cytokeratin 5/6, *EGFR* Epidermal Growth Factor Receptor, *IGF1R* Insulin-like growth factor type 1 receptor

Photographs of slides were taken using an Eclipse 50i microscope and a Nikon DS Fi-1 digital camera (Nikon Instruments Europe B.V.).

### Statistical analysis

The Statview (Statview 5 SAS Institute Inc.) and R (R 3.1.1 GUI 1.65) softwares were used for statistical analyses. Results are given as median and range unless otherwise indicated. Non-parametric tests were used after checking for normality and independence of the data by Kolmogorov-Smirnov test and graphic assessment. The correlation between IGF1R expression and categorical variables (age groups, histological grade, clinical stage, nodal stage, hormone receptor status, and immunophenotype) was analyzed using the Pearson chi-square test or the Fisher exact test. Correlations between numeric variables were determined by Spearman’s test. The Kaplan-Meier non-parametric method was used for univariate survival analysis and the log-rank test was used to assess differences among groups. Cox proportional-hazard regression model was used to examine all factors found to be predictive of survival in univariate analysis simultaneously. A *p*-value of less than 0.05 was considered significant.

## Results

### Clinicopathological findings

The study population consisted in 117 intact and 33 spayed female dogs. Age at surgery ranged from 5.1 to 16.3 years (median 10.9 years). The 150 invasive carcinomas were classified as Luminal and Triple Negative according to ER, PR and HER2 expressions [4, 5]: 47 (31.3 %) were of Luminal subtype (ERα ≥ 10 % and/or PR ≥ 10 %), of which 17 were Luminal-A (Ki-67 < 20 %) and 30 were Luminal-B (Ki-67 ≥ 20 %), and 103 (68.7 %) were classified as Triple Negative (ERα < 10 %, PR < 10 %, HER2 score other than 3+), of which 70 were basal-like (Cytokeratin-CK 5/6 and/or Epidermal Growth Factor Receptor-EGFR positive), and 33 were non-basal-like (CK 5/6 and EGFR negative). No carcinoma was HER2 overexpressing, although immunohistochemical scores 3+ were obtained with the positive controls (human breast cancer lines, control slides provided by Roche Diagnostics). The main clinicopathological findings are summarized in Table 1.

The median follow-up period was 36.3 months. In total, 130 dogs (86.7 %) died. The median time between the date of diagnosis and the date of death was 8.4 months [2 days–60.3 months]. The median DFI was 22.5 months with a 2-year recurrence and/or metastasis rate of 42 %. The median SS was 28.1 months with a 2-year cancer-related mortality rate of 39.3 %. The median OS was 11.0 months with a 2-year mortality rate of 68.7 %.

### IGF1R expression

**Fig. 1:**
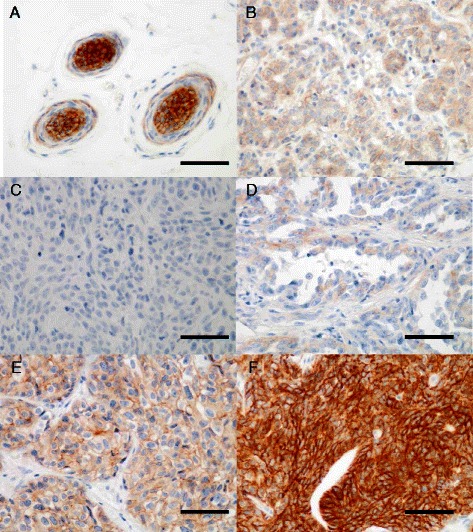
Immunohistochemical staining of IGF1R expression in normal and neoplastic canine mammary glands. IGF1R (Insulin-like growth factor type 1 receptor) expression was scored according to the intensity of the membrane staining in accordance with the HER-2 scoring system. **a** Hair follicle positive for IGF1R expression, **b** Normal mammary gland with a score 2+ for IGF1R, **c** Invasive ductal mammary carcinoma with a score 0 for IGF1R, **d** Invasive ductal mammary carcinoma with a score 1+ for IGF1R, (E) Invasive ductal mammary carcinoma with a score 2+ for IGF1R, **f** Invasive ductal mammary carcinoma with a score 3+ for IGF1R (Immunohistochemical staining, original magnification × 400). Bar = 50 micrometers

The IGF1R staining was exclusively observed in the plasma membrane with cytoplasmic blush only observed when IGF1R was strongly expressed. Only membrane immunoreactivity was taken into account for scoring IGF1R expression. IGF1R was strongly expressed in epithelial cells of the hair follicles, hyperplastic and dysplastic mammary tissues adjacent to the tumors (Fig. 1). The number of cases with IGF1R score 0-1+ was 34 (22.7 %, of which 11 (7.3 %) score 0 and 23 (15.4 %) score 1+), 54 cases (36.0 %) were IGF1R score 2+ and 62 (41.3 %) were IGF1R score 3+ (Fig. 1). Considering the luminal and triple negative immunophenotypes separately, the IGF1R 0–1+, 2+ and 3+ scores occurred in 17 (36.2 %), 14 (29.8 %) and 16 (34.0 %) luminal canine mammary carcinomas and in 17 (16.5 %), 40 (38.8 %) and 46 (44.7 %) triple-negative canine mammary carcinomas respectively.

### Association of IGF1R expression and clinicopathological features

IGF1R overexpression (IHC score 3+) was significantly associated with aggressive features including lymphovascular invasion, histological grade III, absent or low ER and PR expression, and the TN immunophenotype (Table 2). In the Luminal subtype, IGF1R overexpression was also significantly correlated with aggressive features including high histological grade (OR = 7.78 [1.71–45.30], *p* = 0.01) and lymphovascular invasion (OR = 5.42 [1.27–27.20], *p* = 0.03), except for dermal infiltration for which IGF1R score 2+ (OR = 0.07 [0.03–0.46], *p* = 0.02) and 3+ (OR = 0.13 [0.02–0.64], *p* = 0.02) were associated with an absence of dermal infiltration (Additional file 2: Table S2). In the TN subtype, IGF1R overexpression was only significantly related to a high histological grade (OR = 5.54 [1.67–22.25], *p* = 0.02).

**Table 2:** Significant associations between IGF1R expression and clinicopathological features of the 150 canine mammary carcinomas

Parameters	Fisher’s exact test	IGF1R score 2+			IGF1R score 3+		
		*p*-value	OR	95 % CI	*p*-value	OR	95 % CI
Histological grade	<0.001						
Grade I or II		-	1.00	-	-	1.00	-
Grade III		0.007	3.86	1.49–10.99	<0.001	6.54	2.57–18.53
LVI	0.006						
Absent		-	1.00	-	-	1.00	-
Present		0.87	1.08	0.44–2.68	0.01	3.11	1.32–7.62
ER expression	0.004						
Positive (≥ 10 %)		-	1.00	-	-	1.00	-
Negative (< 10 %)		0.003	4.54	1.69–13.01	0.01	3.29	1.32–8.46
PR expression	0.04						
Positive (≥ 10 %)		-	1.00	-	-	1.00	-
Negative (< 10 %)		0.07	2.88	0.93–9.47	0.02	4.10	1.29–14.54
Immunophenotype	0.03						
Luminal		-	1.00	-	-	1.00	-
Triple Negative		0.02	2.86	1.16–7.20	0.02	2.88	1.20–7.04

IGF1R score 0–1+ is considered as the reference for each parameter

*IGF1R* Insulin-like growth factor type 1 receptor, *LVI* Lymphovascular Invasion, *ER* Estrogen Receptor, *PR* Progesterone Receptor, *OR* Odds Ratio, 95 % CI 95 % Confidence Interval

### Prognostic value of IGF1R expression

**Fig. 2:**
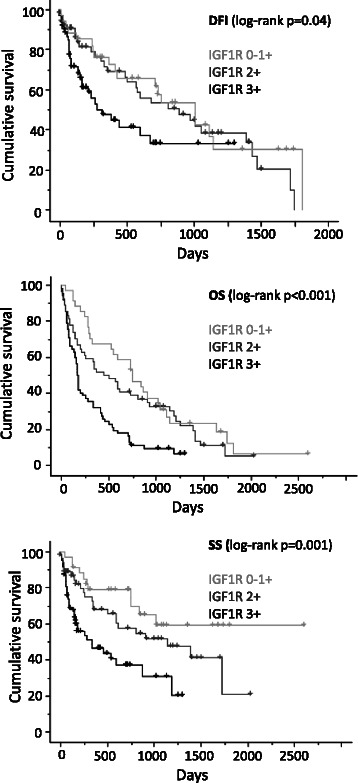
Kaplan-Meier analysis of OS, SS and DFI in 150 canine invasive mammary carcinomas according to IGF1R expression. IGF1R: Insulin-like growth factor type 1 receptor

By univariate analysis, IGF1R overexpression was associated with a poor outcome in terms of disease-free interval (*p* = 0.04), overall (*p* < 0.001) and specific (*p* = 0.001) survival (Fig. 2). Univariate analyses revealed that other factors were associated with a poor prognosis (DFI, OS and SS), including multifocality of the mammary carcinoma, nodal stage at diagnosis, histological grade, surgical margin status, lymphovascular invasion, ER expression and immunophenotype (Tables 3, 4 and 5). Multivariate analysis using Cox proportional-hazard regression was then carried out. When several significant prognostic factors were overlapping (for example nodal stage at mastectomy and lymphovascular invasion or immunophenotype and ER/PR expression), only one was selected as a covariate in the model.

**Table 3:** Factors associated with overall survival (OS) in canine invasive mammary carcinomas (*n* = 150)

Criteria	OS: Univariate analysis			OS: Multivariate analysis		
	(log-rank test) *N* = 150			(Cox regression model) *N* = 150		
	HR	95 % CI	*p*-value	HR	95 % CI	*p*-value
Age			0.005			0.002
<11 yrs	1.00	-		1.00	-	
≥11 yrs	1.66	1.17–2.37		1.79	1.24–2.60	
Multifocality			0.04			0.96
Unifocal	1.00	-		1.00	-	
Multicentric	1.89	1.01–3.54		1.02	0.51–2.04	
Lymph node status			<0.001			
N0	1.00	-		-	-	-
N1	3.53	1.79–6.93				
Histological grade			0.006			0.53
Grade I	1.00	-	-	1.00	-	-
Grade II	1.67	0.93–2.99	0.09	1.33	0.70–2.53	0.38
Grade III	2.37	1.35–4.18	0.003	1.42	0.77–2.62	0.26
Lymphovascular invasion			<0.001			0.01
No LVI	1.00	-		1.00	-	
LVI	2.53	1.78–3.60		1.71	1.14–2.56	
Surgical margins			<0.001			0.006
Complete excision	1.00	-		1.00	-	
Incomplete excision	2.32	1.62–3.33		1.81	1.18–2.75	
Muscle infiltration			0.001			0.77
No	1.00	-		1.00	-	
Yes	1.86	1.27–2.71		1.07	0.70–1.63	
Peritumoral Inflammation			0.02			0.04
No	1.00	-		1.00	-	
Yes	1.53	1.08–2.17		1.53	1.03–2.29	
ER			0.03			
≥ 10 %	1.00	-		-	-	-
< 10 %	1.48	1.04–2.11				
Immunophenotype			0.03			0.17
Luminal	1.00	-		1.00	-	
Triple negative	1.54	1.05–2.26		1.34	0.88–2.04	
IGF1R			<0.001			0.002
weak (0–1+)	1.00	-	-	1.00	-	-
moderate (2+)	1.31	0.81–2.10	0.27	1.40	0.85–2.33	0.19
strong (3+)	2.62	1.63–4.20	<0.001	2.74	1.63–4.62	0.002

Univariate (log rank test) and multivariate survival analyses (Cox proportional hazard regression)

*HR* Hazard Ratio, 95 % CI 95 % Confidence Interval, *ER* Estrogen Receptor, *IGF1R* Insulin-like Growth Factor type 1 Receptor, *LVI* Lymphovascular Invasion

When several significant prognostic factors overlapped, only one was selected for the multivariate analysis (LVI was chosen between lymph node status and LVI because it could have been determined in all cases and immunophenotype was preferred to ER expression)

**Table 4:** Factors associated with specific survival (SS) in canine invasive mammary carcinomas (*n* = 150)

Criteria	SS: Univariate analysis			SS: Multivariate analysis		
	(log-rank test) *N* = 150			(Cox regression model) *N* = 150		
	HR	95 % CI	*p*-value	HR	95 % CI	*p*-value
Body size			0.04			0.63
> 10 kgs	1.00	-		1.00		
≤ 10 kgs	1.68	1.01–2.78		1.16	0.64–2.09	
Multifocality			0.02			0.85
Unifocal	1.00	-		1.00	-	
Multicentric	2.36	1.12–4.97		1.09	0.44–2.72	
Lymph node status			0.001			
N0	1.00	-		-	-	-
N1	5.07	1.88–13.67				
Histological grade			0.01			0.44
Grade I	1.00	-		1.00	-	-
Grade II	2.52	0.97–6.59	0.06	2.02	0.66–6.23	0.22
Grade III	3.74	1.47–9.55	0.006	2.03	0.67–6.19	0.21
Lymphovascular invasion			<0.001			0.002
No LVI	1.00	-		1.00	-	
LVI	4.48	2.68–7.51		2.66	1.43–4.94	
Surgical margins			<0.001			0.24
Complete excision	1.00	-		1.00	-	
Incomplete excision	2.34	1.43–3.83		1.46	0.77–2.78	
Muscle infiltration			0.01			0.76
No	1.00	-		1.00	-	
Yes	1.88	1.14–3.11		1.10	0.58–2.11	
Peritumoral Inflammation			0.04			0.07
No	1.00	-		1.00	-	
Yes	1.64	1.02–2.63		1.74	0.96–3.15	
Central necrosis			0.03			0.005
No	1.00	-		1.00	-	
Yes	0.57	0.34–0.96		0.43	0.24–0.78	
ER			0.04			
≥ 10 %	1.00	-		-	-	-
< 10 %	1.91	1.02–3.56				
Ki-67			0.01			0.29
< 20 %	1.00			1.00	-	
≥ 20 %	2.65	1.21–5.80		1.70	0.64–4.51	
Immunophenotype			0.004			0.06
Luminal	1.00	-		1.00	-	
Triple negative	2.35	1.31–4.22		1.94	0.96–3.88	
IGF1R			0.001			0.03
weak (0–1+)	1.00	-	-	1.00	-	-
moderate (2+)	1.68	0.82–3.44	0.15	1.66	0.72–3.85	0.24
strong (3+)	3.36	1.68–6.72	<0.001	2.81	1.25–6.31	0.01

Univariate (log rank test) and multivariate survival analyses (Cox proportional hazard regression)

*HR* Hazard Ratio, 95 % CI 95 % Confidence Interval, *ER* Estrogen Receptor, *IGF1R* Insulin-like Growth Factor type 1 Receptor, *LVI* Lymphovascular Invasion

When several significant prognostic factors overlapped, only one was selected for the multivariate analysis (LVI was chosen between lymph node status and LVI because it could have been determined in all cases and immunophenotype was preferred to ER expression)

**Table 5:** Factors associated with disease-free interval (DFI) in canine invasive mammary carcinomas (*n* = 150)

Criteria	DFI: Univariate analysis			DFI: Multivariate analysis		
	(log-rank test) *N* = 150			(Cox regression model) *N* = 150		
	HR	95 % CI	*p*-value	HR	95 % CI	*p*-value
Body size			0.005			0.07
> 10 kgs	1.00	-		1.00		
≤ 10 kgs	1.99	1.23–3.21		1.66	0.95–2.90	
Age			0.007			0.02
<11 yrs	1.00	-		1.00	-	
≥11 yrs	1.88	1.19–2.98		1.91	1.13–3.24	
Multifocality			0.01			0.76
Unifocal	1.00	-		1.00	-	
Multicentric	2.56	1.22–5.38		0.86	0.32–2.29	
Histological grade			0.06			0.46
Grade I	1.00	-	-	1.00	-	
Grade II	1.90	0.89–4.04	0.10	1.76	0.67–4.62	0.25
Grade III	2.43	1.17–5.06	0.02	1.83	0.69–4.86	0.22
Lymphovascular invasion			<0.001			0.04
No LVI	1.00	-		1.00	-	
LVI	2.86	1.81–4.51		1.91	1.02–3.58	
Surgical margins			0.005			0.15
Complete excision	1.00	-		1.00	-	
Incomplete excision	1.94	1.22–3.07		1.57	0.85–2.88	
Muscle infiltration			0.005			0.99
No	1.00	-		1.00	-	
Yes	2.00	1.23–3.28		1.00	0.54–1.86	
Peritumoral Inflammation			0.04			0.07
No	1.00	-		1.00	-	
Yes	1.57	1.00–2.47		1.69	0.95–2.99	
Central necrosis			0.03			0.04
No	1.00	-		1.00	-	
Yes	0.56	0.34–0.93		0.52	0.28–0.97	
Immunophenotype			0.02			0.18
Luminal	1.00	-		1.00	-	
Triple negative	1.80	1.08–3.00		1.54	0.82–2.86	
CK5/6			0.01			0.09
< 10 %	1.00	-		1.00	-	
≥ 10 %	0.55	0.35–0.87		0.61	0.34–1.08	
IGF1R			0.04			0.23
weak (0–1+)	1.00	-	-	1.00	-	-
moderate (2+)	1.22	0.68–2.19	0.51	1.15	0.58–2.28	0.70
strong (3+)	2.09	1.14–3.83	0.02	1.74	0.86–3.55	0.13

Univariate (log rank test) and multivariate survival analyses (Cox proportional hazard regression)

*HR* Hazard Ratio, 95 % CI 95 % Confidence Interval, *CK5/6* Cytokeratin 5/6, *IGF1R* Insulin-like Growth Factor type 1 Receptor, *LVI* Lymphovascular Invasion

For overall survival, IGF1R overexpression appeared to be a strong and independent prognostic factor associated with a poor outcome, as well as an age of more than 11 years, lymphovascular invasion, positive margin status of the surgical sample and the presence of a peritumoral inflammation (Table 3). With regard to specific survival, IGF1R overexpression, lymphovascular invasion, and the presence of central necrosis showed a significant independent prognostic value (Table 4). By multivariate analysis for disease-free interval, IGF1R overexpression was no longer significantly associated with an earlier recurrence, new primary tumor and/or lymph node and distant metastasis (*p* = 0.13) (Table 5).

The prognostic impact of IGF1R was also assessed separately in the luminal and the TN immunophenotypes.

In the luminal subtype (*n* = 47), IGF1R overexpression was associated with a shorter OS (HR = 3.13 [1.41–6.96]; *p* = 0.005) and SS (HR = 4.72 [1.42–15.77]; *p* = 0.01) by univariate analysis (Additional file 3: Tables S3 and Additional file 4: Table S4). By multivariate analysis, IGF1R overexpression was also a significant strong and independent prognostic factor associated with a poor outcome in terms of OS and SS, as well as an age of more than 11 years.

In the TN subtype (*n* = 103), IGF1R overexpression was also associated with a shorter OS (HR = 2.24 [1.23–4.10]; *p* = 0.009) and SS (HR = 2.49 [1.07–5.81]; *p* = 0.03) by univariate analysis. IGF1R expression retained a significant and independent prognostic value for OS by multivariate analysis, as well as the age of the dog at neutering, occurrence of a new primary mammary tumor, histological grade, surgical margin status and presence of central necrosis (Additional file 5: Table S5). Finally, IGF1R was also a significant and independent prognostic factor for SS in the TN immunophenotype, with lymphovascular invasion and central necrosis (HR = 0.47 [0.24–0.93]; *p* = 0.03) as covariates (Additional file 6: Table S6).

IGF1R expression did not show any prognostic value in terms of DFI either in the luminal or TN subgroup.

## Discussion

The objective of this study was to investigate IGF1R expression in a large cohort of canine invasive carcinomas, focusing on its relationship with the clinicopathological features and prognosis, in terms of overall, specific and disease-free survivals, in order to evaluate the similarities between the role of IGF1R in the canine species and those previously reported in human breast cancer. We found that IGF1R was frequently expressed in canine invasive mammary carcinoma, as more than 90 % showed at least a weak membrane staining for IGF1R. This result is in accordance with the previous human studies, as usually more than 80 % of the invasive breast cancer cells are positive for IGF1R [18, 35, 38]. In human breast cancer, few studies take into account both membrane and cytoplasmic IGF1R expression [18, 39, 40]. We only considered membrane staining for scoring IGF1R expression, as cytoplasmic blush was only observed when IGF1R was strongly expressed. Methods used for IGF1R scoring depend on the study, but most of the published results consider that a score of 3+ by immunohistochemistry (mostly defined as complete and intense membrane staining in more than 10 % of the cells, as for HER2 scoring) defined IGF1R overexpression [19, 35]. Thus, we chose to score IGF1R in accordance with the scoring of HER2 in breast cancer and then grouped the negative scores (complete absence of membrane staining or the presence of weak membrane staining in less than 10 % of the cells) and 1+ (incomplete membrane staining in more than 10 % of the cells), compared with the positive scores 2+ (complete and weak to moderate membrane staining in more than 10 % of the cells) and 3+ (complete and intense membrane staining in more than 10 % of the cells) as Shin *et al.* previously did in human breast cancer [19]. However, the grouping of the score 0 and 1+ is questionable, as the normal canine mammary gland [22] (Fig. 1), like the human breast [41, 42], naturally shows a weak (1+) to moderate (2+) IGF1R expression, implying that the absence of expression is abnormal and not necessarily a good prognostic factor. Indeed, some studies showed that IGF1R negativity and down-regulation was associated with a worse prognosis [43] in tamoxifen-treated postmenopausal breast cancer and correlated with aggressive features such as poor differentiation and high proliferation [44]. The number of cases in the present study with a score 0 for IGF1R expression was too small (*n* = 11) to analyze this group separately, implying that this is a rare condition that requires more cases for definitive conclusions.

When luminal and triple-negative subtypes were assessed separately, IGF1R overexpression (score 3+) was comparable in frequency to that reported in human breast cancer in which more than 45 % of the triple-negative breast carcinomas show strong expression of IGF1R [18, 19, 40, 41]. In human breast cancer and canine mammary carcinoma, several studies have shown that IGF1R expression parallels ER expression [18, 20, 39, 41], but we found that IGF1R overexpression was correlated with the negativity for ER and PR in the total cohort as Law *et al.* showed for phosphorylated IGF1R/IR expression in human breast cancer [45]. This contradictory result could be due to a biological difference concerning IGF1R and ER between dogs and humans. The fact that IGF1R parallels ER expression in canine mammary carcinoma in the study of Queiroga *et al.* [20] is also controversial: the cohort was small (40 mammary carcinomas) and unlike the present study, the invasive nature of the mammary carcinomas was not assessed. In our luminal subgroup, no correlation was found between hormonal receptor (ER and PR) and IGF1R expression. Nevertheless, this result has to be confirmed on a larger cohort of luminal canine mammary carcinomas.

IGF1R expression was also correlated with other aggressive features in both luminal and TN subtypes (such as high histological grades or presence of lymphovascular invasion). These results are in accordance with previously published studies in canine mammary carcinoma, as IGF-1 and IGF1R expression were respectively related to tumor malignancy [20] and histological types with worse prognosis [21]. This finding is in line with the fact that IGF1R is considered as a real oncogene closely involved in survival, proliferation, tumor growth, invasion and metastasis as it was demonstrated in canine osteosarcoma-derived cell lines [23]. In human breast cancer, results are controversial and generally depend on the ER status of the carcinomas. Indeed, extensive crosstalk between ER and IGF1R is now well-established from several *in vitro* studies, which demonstrate a synergistic effect of IGF1R and ER on the proliferation of human breast cancer cells [46, 47]. Even if some studies did not find any significant results [35, 48, 49], IGF1R positivity was generally related to favorable prognostic features in ER-positive breast cancer, including low histological grade [19]. On the contrary, strong IGF1R expression was associated with aggressive features in triple negative breast cancer, such as high histological grade [40]. However, no study to date has investigated the crosstalk between IGF1R and ER in canine mammary cell lines. A difference of receptor biology between Human and Dog cannot be excluded and should thus be further investigated.

Some studies show that the complete negativity or low expression of IGF1R is related to a worse prognosis [43, 44]. On the contrary, rare studies reveal that high IGF1R mRNA [50] and phosphorylated IGF1R/IR [45] are associated with a poor prognosis, whatever the molecular subtype of breast cancer. In addition, even if human studies show contradictory results, it seems that the IGF1R prognostic value also depends on the tumor ER status: in ER-positive mammary carcinomas, IGF1R overexpression is related to a favorable prognosis [18, 19] as opposed to the triple-negative subtype, in which IGF1R overexpression is associated with a poor outcome [18, 19, 40]. In the present study, no difference was found between the luminal and triple-negative subtypes of canine mammary carcinoma according to the prognostic value of IGF1R expression: IGF1R overexpression was associated with a poor prognosis in both luminal and triple-negative canine mammary carcinomas. The fact that none of the dogs of this study received adjuvant endocrine therapy is however a major difference between humans and dogs after a diagnosis of luminal mammary carcinoma, and this difference is likely to interfere with prognosis. Furthermore, only 47 luminal mammary carcinomas were included in this study and further investigations with a higher number of luminal mammary carcinomas are needed to confirm this result. Nonetheless, the expression and prognostic value of IGF1R overexpression is of particular interest in the triple negative subtype since it is associated with a poor prognosis, particularly in young women for which this type is more frequent [51]. Indeed, there is a lack of effective treatment for triple negative breast cancer and the search for relevant therapeutic targets is of major concern [52]. IGF1R could be a good candidate [53] with a translational approach based on clinical trials in dogs.

## Conclusions

IGF1R overexpression is common in canine mammary carcinoma and related to a poor clinical outcome, particularly in the triple negative subtype. The Dog appears to be a relevant naturally-occurring model of IGF1R overexpressing triple-negative breast cancer, opening the way for possible translational perspectives in the search for new therapeutic opportunities, including anti-IGF1R therapies.

## Additional files

Additional file 1: Table S1. Primary antibodies and immunohistochemical protocols (Benchmark XT Ventana, Roche Diagnostics). All dilutions were performed using a commercially available diluent (Ventana Medical Systems). ^a^Ultraview and ^b^Optiview Universal DAB detection kit: multimer-technology based detection system. ^c^IView Universal DAB detection kit: biotin streptavidin system. ERα: Estrogen Receptor alpha, PR: Progesterone Receptor, HER2: Epidermal Growth Factor type 2 Receptor, CK5/6: Cytokeratin 5/6, EGFR: Epidermal Growth Factor type 1 Receptor, IGF1R: Insulin-like growth factor type 1 receptor. CC1: Cell Conditioning 1. (DOC 33 kb)
Additional file 2: Table S2. Significant associations between IGF1R expression and clinicopathological features of 47 luminal canine mammary carcinomas. IGF1R score 0–1+ is considered as the reference for each parameter. IGF1R Insulin-like Growth Factor type 1 Receptor. LVI: Lymphovascular Invasion. OR: Odd Ratio. 95 % CI: 95 % Confidence Interval. (DOC 30 kb)
Additional file 3: Table S3. Factors associated with overall survival (OS) in 47 Luminal canine invasive mammary carcinomas. Univariate (log rank test) and multivariate survival analyses (Cox proportional hazard regression). HR: Hazard Ratio, 95 % CI: 95 % Confidence Interval, HER2: Epidermal Growth Factor type 2 Receptor, CK5/6: Cytokeratin 5/6, EGFR: Epidermal Growth Factor type 1 Receptor, IGF1R: Insulin-like Growth Factor type 1 Receptor. (DOC 35 kb)
Additional file 4: Table S4. Factors associated with specific survival (SS) in 47 Luminal canine invasive mammary carcinomas. Univariate (log rank test) and multivariate survival analyses (Cox proportional hazard regression). HR: Hazard Ratio, 95 % CI: 95 % Confidence Interval, HER2: Epidermal Growth Factor type 2 Receptor, IGF1R: Insulin-like Growth Factor type 1 Receptor, LVI: Lymphovascular Invasion. (DOC 33 kb)
Additional file 5: Table S5. Factors associated with overall survival (OS) in 103 Triple-negative canine invasive mammary carcinomas. Univariate (log rank test) and multivariate survival analyses (Cox proportional hazard regression). HR: Hazard Ratio, 95 % CI: 95 % Confidence Interval, IGF1R: Insulin-like Growth Factor type 1 Receptor, LVI: Lymphovascular Invasion. When several significant prognostic factors overlapped, only one was selected for the multivariate analysis (LVI was chosen between lymph node status and LVI because it could have been determined in all cases). (DOC 38 kb)
Additional file 6: Table S6. Factors associated with specific survival (SS) in 103 Triple-negative canine invasive mammary carcinomas. Univariate (log rank test) and multivariate survival analyses (Cox proportional hazard regression). HR: Hazard Ratio, 95 % CI: 95 % Confidence Interval, IGF1R: Insulin-like Growth Factor type 1 Receptor, LVI: Lymphovascular Invasion. When several significant prognostic factors overlapped, only one was selected for the multivariate analysis (LVI was chosen between lymph node status and LVI because it could have been determined in all cases). (DOC 35 kb)

## Abbreviations

CK5/6: Cytokeratin 5/6
CMC: Canine mammary carcinoma
DFI: Disease-free interval
EGFR: Epidermal growth factor receptor
ER: Estrogen receptor
HE: Hematoxylin and eosin
HER2: Human epidermal growth factor receptor 2
IGF: Insulin-like growth factor
IGF1R: Insulin like growth factor type 1 receptor
IHC: Immunohistochemistry
IR: Insulin receptor
OS: Overall survival
PR: Progesterone receptor
SS: Specific survival
TN: Triple negative
