# Supplementary material for: The dog as a naturally-occurring model for insulin-like growth factor type 1 receptor-overexpressing breast cancer: an observational cohort study
Source: BMC Cancer. 2015 Oct 8;15:664. doi: 10.1186/s12885-015-1670-6 (PMC4598970; doi:10.1186/s12885-015-1670-6)
Supplement: Additional file 1: Table S1. — Primary antibodies and immunohistochemical protocols (Benchmark XT Ventana, Roche Diagnostics). All dilutions were performed using a commercially available diluent (Ventana Medical Systems). aUltraview and bOptiview Universal DAB detection kit: multimer-technology based detection system. cIView Universal DAB detection kit: biotin streptavidin system. ERα: Estrogen Receptor alpha, PR: Progesterone Receptor, HER2: Epidermal Growth Factor type 2 Receptor, CK5/6: Cytokeratin 5/6, EGFR: Epidermal Growth Factor type 1 Receptor, IGF1R: Insulin-like growth factor type 1 receptor. CC1: Cell Conditioning 1. (DOC 33 kb) [file 12885_2015_1670_MOESM1_ESM.doc]

|  | **ERα** | **PR** | **HER2** | **Ki67** | **CK5/6** | **EGFR** | **IGF1R** |
| --- | --- | --- | --- | --- | --- | --- | --- |
| **Primary antibody** | Monoclonal mouse anti-human  Clone C311 (Santa Cruz)  Dilution 1:50 | Monoclonal rabbit anti-human  Clone PR1E2 (Roche Diagnostics)  Prediluted | Monoclonal rabbit anti-human  Clone 4B5 (Roche Diagnostics)  Prediluted | Monoclonal mouse anti-human  Clone MIB-1 (Dako)  Dilution 1:50 | Monoclonal mouse anti-human  Clone D5/16 B4 (Dako)  Dilution 1:50 | Monoclonal mouse anti-human  Clone 31G7 (Invitrogen)  Dilution 1:20 | Monoclonal rabbit anti-human  Clone G11 (Roche Diagnostics)  Dilution 1:4 |
| **Incubation time** | 44 min | 44 min | 8 min | 32 min | 16 min | 32 min | 32 min |
| **Universal DAB Detection kit** | Iview^c^ | Iview^c^ | Ultraview^a^ | Iview^c^ | Iview^c^ | Iview^c^ | Optiview^b^ |
| **Secondary antibody** | Polyclonal goat anti-mouse IgG (Dako)  Dilution 1:300 | Polyclonal goat anti-rabbit Ig (Dako)  Dilution 1:100 | - | Polyclonal goat anti-mouse IgG (Dako)  Dilution 1:300 | Polyclonal goat anti-mouse IgG (Dako)  Dilution 1:100 | Polyclonal goat anti-mouse IgG (Dako)  Dilution 1:100 | - |
| **Antigen Retrieval** | None | 90 min (95°C-CC1) | 30 min (95°C-CC1) | 60 min (95°C-CC1) | 30 min (95°C-CC1) | 20 min (protease-1) | 32 min (95°C-CC1) |
| **Positive control** | Canine normal mammary gland | Canine normal mammary gland | Pathway Her-2 4in1 control slides | Canine skin and lymph node | Canine skin | Canine skin | Canine skin |

**Supplementary Table 1: Primary antibodies and immunohistochemical protocols (Benchmark XT Ventana, Roche Diagnostics).** All dilutions were performed using a commercially available diluent (Ventana Medical Systems)**.** ^a^Ultraview and ^b^Optiview Universal DAB detection kit: multimer-technology based detection system. ^c^IView Universal DAB detection kit: biotin streptavidin system. ERα: Estrogen Receptor alpha, PR: Progesterone Receptor, HER2: Epidermal Growth Factor type 2 Receptor, CK5/6: Cytokeratin 5/6, EGFR: Epidermal Growth Factor type 1 Receptor, IGF1R: Insulin-like growth factor type 1 receptor. CC1: Cell Conditioning 1.
