# Supplementary material for: The dog as a naturally-occurring model for insulin-like growth factor type 1 receptor-overexpressing breast cancer: an observational cohort study
Source: BMC Cancer. 2015 Oct 8;15:664. doi: 10.1186/s12885-015-1670-6 (PMC4598970; doi:10.1186/s12885-015-1670-6)
Supplement: Additional file 2: Table S2. — Significant associations between IGF1R expression and clinicopathological features of 47 luminal canine mammary carcinomas. IGF1R score 0–1+ is considered as the reference for each parameter. IGF1R Insulin-like Growth Factor type 1 Receptor. LVI: Lymphovascular Invasion. OR: Odd Ratio. 95 % CI: 95 % Confidence Interval. (DOC 30 kb) [file 12885_2015_1670_MOESM2_ESM.doc]

| **Parameters** | **Fisher’s exact test** | **IGF1R score 2+** | | | **IGF1R score 3+** | | |
| --- | --- | --- | --- | --- | --- | --- | --- |
|  |  | **p-value** | **OR** | **95% CI** | **p-value** | **OR** | **95% CI** |
| **Histological grade**  Grade I or II  Grade III | **0.03** | -  0.13 | 1.00  3.50 | -  0.72-20.49 | -  **0.01** | 1.00  **7.78** | -  **1.71- 45.30** |
| **LVI**  Absent  Present | **0.01** | -  0.52 | 1.00  0.54 | -  0.07-3.32 | -  **0.03** | 1.00  **5.42** | -  **1.27-27.20** |
| **Dermal Infiltration**  Absent  Present | **0.009** | -  **0.02** | 1.00  **0.07** | -  **0.03-0.46** | -  **0.02** | 1.00  **0.13** | -  **0.02-0.64** |

**Supplementary Table 2: Significant associations between IGF1R expression and clinicopathological features of 47 luminal canine mammary carcinomas. IGF1R score 0-1+ is considered as the reference for each parameter.**

IGF1R Insulin-like Growth Factor type 1 Receptor. LVI: Lymphovascular Invasion. OR: Odd Ratio. 95% CI: 95% Confidence Interval
