# Supplementary material for: The dog as a naturally-occurring model for insulin-like growth factor type 1 receptor-overexpressing breast cancer: an observational cohort study
Source: BMC Cancer. 2015 Oct 8;15:664. doi: 10.1186/s12885-015-1670-6 (PMC4598970; doi:10.1186/s12885-015-1670-6)
Supplement: Additional file 3: Table S3. — Factors associated with overall survival (OS) in 47 Luminal canine invasive mammary carcinomas. Univariate (log rank test) and multivariate survival analyses (Cox proportional hazard regression). HR: Hazard Ratio, 95 % CI: 95 % Confidence Interval, HER2: Epidermal Growth Factor type 2 Receptor, CK5/6: Cytokeratin 5/6, EGFR: Epidermal Growth Factor type 1 Receptor, IGF1R: Insulin-like Growth Factor type 1 Receptor. (DOC 35 kb) [file 12885_2015_1670_MOESM3_ESM.doc]

| **Criteria** | **OS: Univariate analysis**  **(Log-rank test) N=47** | | | **OS: Multivariate analysis**  **(Cox regression model) N=47** | | |
| --- | --- | --- | --- | --- | --- | --- |
|  | **HR** | **95% CI** | **p-value** | **HR** | **95% CI** | **p-value** |
| **Age**  <11 yrs  ≥11 yrs | 1.00  **3.60** | -  **1.71-7.59** | **<0.001** | 1.00  **6.87** | -  **2.63-17.87** | **<0.001** |
| **Muscular infiltration**  No  Yes | 1.00  **2.75** | -  **1.21-6.25** | **0.02** | 1.00  0.98 | **-**  0.38-2.56 | 0.97 |
| **HER2**  Score 2+  Score 1+  Score 0 | 1.00  **0.32**  **0.27** | -  **0.12-0.89**  **0.10-0.74** | **0.03**  -  **0.03**  **0.01** | 1.00  0.38  0.70 | -  0.11-1.30  0.18-2.79 | 0.16  -  0.12  0.61 |
| **CK5/6**  <10%  ≥10% | 1.00  **0.32** | -  **0.15-0.68** | **0.003** | 1.00  0.50 | -  0.16-1.55 | 0.23 |
| **EGFR**  <10%  ≥10% | 1.00  **2.88** | **-**  **1.37-6.09** | **0.006** | 1.00  2.73 | -  0.96-7.80 | 0.06 |
| **IGF1R**  weak (0-1+)  moderate (2+)  strong (3+) | 1.00  1.17  **3.13** | **-**  0.50-2.72  **1.41-6.96** | **0.01**  -  0.71  **0.005** | 1.00  1.67  **12.12** | -  0.64-4.38  **3.76-39.14** | **<0.001**  -  0.30  **<0.001** |

**Supplementary Table 3: Factors associated with overall survival (OS) in 47 Luminal canine invasive mammary carcinomas. Univariate (log rank test) and multivariate survival analyses (Cox proportional hazard regression).** HR: Hazard Ratio, 95% CI: 95% Confidence Interval, HER2: Epidermal Growth Factor type 2 Receptor, CK5/6: Cytokeratin 5/6, EGFR: Epidermal Growth Factor type 1 Receptor, IGF1R: Insulin-like Growth Factor type 1 Receptor.
