# Supplementary material for: The dog as a naturally-occurring model for insulin-like growth factor type 1 receptor-overexpressing breast cancer: an observational cohort study
Source: BMC Cancer. 2015 Oct 8;15:664. doi: 10.1186/s12885-015-1670-6 (PMC4598970; doi:10.1186/s12885-015-1670-6)
Supplement: Additional file 4: Table S4. — Factors associated with specific survival (SS) in 47 Luminal canine invasive mammary carcinomas. Univariate (log rank test) and multivariate survival analyses (Cox proportional hazard regression). HR: Hazard Ratio, 95 % CI: 95 % Confidence Interval, HER2: Epidermal Growth Factor type 2 Receptor, IGF1R: Insulin-like Growth Factor type 1 Receptor, LVI: Lymphovascular Invasion. (DOC 33 kb) [file 12885_2015_1670_MOESM4_ESM.doc]

| **Criteria** | **SS: Univariate analysis**  **(Log-rank test) N=47** | | | **SS: Multivariate analysis**  **(Cox regression model) N=47** | | |
| --- | --- | --- | --- | --- | --- | --- |
|  | **HR** | **95% CI** | **p-value** | **HR** | **95% CI** | **p-value** |
| **Age**  <11 yrs  ≥11 yrs | 1.00  **4.44** | -  **1.23-15.98** | **0.02** | 1.00  **10.86** | -  **2.25-52.49** | **0.003** |
| **Lymphovascular invasion**  No LVI  LVI | 1.00  **3.72** | -  **1.28-10.80** | **0.02** | 1.00  1.25 | -  0.31-5.13 | 0.76 |
| **Squamous differentiation**  No  Yes | 1.00  **0.13** | -  **0.02-0.96** | **0.04** | 1.00  0.16 | **-**  0.01-1.97 | 0.15 |
| **Central necrosis**  No  Yes | 1.00  **0.33** | **-**  **0.11-0.95** | **0.04** | 1.00  0.67 | -  0.17-2.70 | 0.58 |
| **HER2**  Score 2+  Score 1+  Score 0 | 1.00  0.26  **0.20** | -  0.07-1.00  **0.05-0.77** | 0.05  -  0.05  **0.02** | 1.00  0.19  1.20 | -  0.03-1.27  0.17-8.54 | 0.15  -  0.09  0.86 |
| **IGF1R**  weak (0-1+)  moderate (2+)  strong (3+) | 1.00  0.35  **4.72** | **-**  0.04-3.14  **1.42-15.77** | **0.006**  -  0.35  **0.01** | 1.00  0.55  **16.30** | -  0.06-5.17  **2.30-115.48** | **0.01**  -  0.61  **0.005** |

**Supplementary Table 4: Factors associated with specific survival (SS) in 47 Luminal canine invasive mammary carcinomas. Univariate (log rank test) and multivariate survival analyses (Cox proportional hazard regression).** HR: Hazard Ratio, 95% CI: 95% Confidence Interval, HER2: Epidermal Growth Factor type 2 Receptor, IGF1R: Insulin-like Growth Factor type 1 Receptor, LVI: Lymphovascular Invasion.
