# Supplementary material for: The dog as a naturally-occurring model for insulin-like growth factor type 1 receptor-overexpressing breast cancer: an observational cohort study
Source: BMC Cancer. 2015 Oct 8;15:664. doi: 10.1186/s12885-015-1670-6 (PMC4598970; doi:10.1186/s12885-015-1670-6)
Supplement: Additional file 5: Table S5. — Factors associated with overall survival (OS) in 103 Triple-negative canine invasive mammary carcinomas. Univariate (log rank test) and multivariate survival analyses (Cox proportional hazard regression). HR: Hazard Ratio, 95 % CI: 95 % Confidence Interval, IGF1R: Insulin-like Growth Factor type 1 Receptor, LVI: Lymphovascular Invasion. When several significant prognostic factors overlapped, only one was selected for the multivariate analysis (LVI was chosen between lymph node status and LVI because it could have been determined in all cases). (DOC 38 kb) [file 12885_2015_1670_MOESM5_ESM.doc]

| **Criteria** | **OS: Univariate analysis**  **(Log-rank test) N=103** | | | **OS: Multivariate analysis**  **(Cox regression model) N=103** | | |
| --- | --- | --- | --- | --- | --- | --- |
|  | **HR** | **95% CI** | **p-value** | **HR** | **95% CI** | **p-value** |
| **Age of neutering**  Ovariectomy after mastectomy  Ovariectomy before mastectomy  Intact female | 1.00  **3.98**  **3.32** | -  **1.44-11.00**  **1.32-8.38** | **0.03**  -  **0.008**  **0.01** | 1.00  **6.66**  **5.27** | -  **2.00-22.09**  **1.66-16.76** | **0.008**  -  **0.002**  **0.005** |
| **Other mammary tumor**  Yes  No | 1.00  **2.15** | -  **1.11-4.17** | **0.02** | 1.00  **3.55** | -  **1.69-7.49** | **<0.001** |
| **Lymph node status**  N0  N1 | 1.00  **4.87** | -  **1.92-12.38** | **<0.001** | - | **-** | - |
| **Histological grade**  Grade I  Grade II  Grade III | 1.00  **2.73**  **3.13** | -  **1.20-6.24**  **1.41-6.99** | **0.02**  -  **0.002**  **0.005** | 1.00  **2.85**  **2.64** | -  **1.12-7.28**  **1.07-6.52** | 0.08  -  **0.03**  **0.03** |
| **Muscle infiltration**  No  Yes | 1.00  **1.57** | -  **1.02-2.41** | **0.04** | 1.00  1.31 | **-**  0.77-2.14 | 0.34 |
| **Lymphovascular invasion**  No LVI  LVI | 1.00  **2.69** | -  **1.75-4.15** | **<0.001** | 1.00  1.63 | -  0.99-2.67 | 0.05 |
| **Surgical margins**  Complete excision  Incomplete excision | 1.00  **2.59** | **-**  **1.69-3.98** | **<0.001** | 1.00  **2.43** | -  **1.40-4.22** | **0.002** |
| **Peritumoral Inflammation**  No  Yes | 1.00  **1.74** | **-**  **1.15-2.64** | **0.009** | 1.00  1.15 | -  0.69-1.91 | 0.60 |
| **Central necrosis**  No  Yes | 1.00  **0.57** | **-**  **0.34-0.98** | **0.04** | 1.00  **0.36** | **-**  **0.19-0.66** | **0.001** |
| **IGF1R**  weak (0-1+)  moderate (2+)  strong (3+) | 1.00  1.22  **2.24** | **-**  0.67-2.25  **1.23-4.10** | **0.008**  -  0.52  **0.009** | 1.00  1.15  2.03 | -  0.56-2.35  0.99-4.18 | **0.04**  -  0.70  0.05 |

**Supplementary Table 5: Factors associated with overall survival (OS) in 103 Triple-negative canine invasive mammary carcinomas. Univariate (log rank test) and multivariate survival analyses (Cox proportional hazard regression).** HR: Hazard Ratio, 95% CI: 95% Confidence Interval, IGF1R: Insulin-like Growth Factor type 1 Receptor, LVI: Lymphovascular Invasion. When several significant prognostic factors overlapped, only one was selected for the multivariate analysis (LVI was chosen between lymph node status and LVI because it could have been determined in all cases)
